# Estimation of DENV-2 Transmission as a Function of Site-Specific Entomological Parameters from Three Cities in Colombia

**DOI:** 10.5334/aogh.2339

**Published:** 2019-03-13

**Authors:** Víctor Hugo Peña-García, Irma Sánchez-Vargas, Rebecca Christofferson, William C. Black, Sair Arboleda, Omar Triana-Chávez

**Affiliations:** 1 Grupo de Biología y Control de Enfermedades Infecciosas (BCEI), Sede de investigaciones universitarias (SIU), Universidad de Antioquia, Medellín, CO; 2 Arthropod-Borne and Infectious Disease Laboratory (AIDL), Department of Microbiology, Immunology and Pathology, Fort Collins, Colorado, US; 3 Department of Pathobiological Sciences, School of Veterinary Medicine, Louisiana State University, Baton Rouge, Louisiana, US

## Abstract

**Background:**

Measuring dengue virus transmission in endemic areas is a difficult task as many variables drive transmission, and often are not independent of one another.

**Objectives:**

We aimed to determine the utility of vectorial capacity to explain the observed dengue infection rates in three hyperendemic cities in Colombia, and tested hypotheses related to three variables: mosquito density, effective vector competence, and biting rate.

**Methods:**

We estimated two of the most influential entomological variables related to cumulative vectorial capacity, which is a modification of the traditional vectorial capacity equation, of three Colombian mosquito populations. Laboratory studies were undertaken to measure vector competence and man biting rate of local mosquito populations. In addition, the assessment of cumulative vectorial capacity also incorporated site-specific estimations of mosquito density and the probability of daily survival from previous studies conducted in those cities.

**Findings:**

We found that the biting rates and mosquito infection rates differed among populations of mosquitoes from these three cities, resulting in differences in the site-specific measures of transmission potential. Specifically, we found that using site-specific entomological measures to populate the cumulative vectorial capacity equation was best at recapitulating observed mosquito infection rates when mosquito density was discounted compared to when we incorporated site-specific density measures.

**Conclusions:**

Specific mosquito-biting rate is likely sufficient to explain transmission differences in these three cities, confirming that this parameter is a critical parameter when predicting and assessing dengue transmission in three Colombian cities with different field observed transmission patterns.

## Introduction

Dengue virus (DENV) is a mosquito-borne virus that circulates in more than 100 countries around the world, [1] and it is estimated that 390 million new infections of DENV occur per year, of which 96 million present with clinical manifestations [2]. DENV is a positive sense-RNA virus belonging to Flaviviridae family and is comprised of four serotypes denoted DENV [1,2,3,4]. When multiple serotypes circulate in the same geographical area is known as hyperendemicity [3,4], and several regions of Colombia have been hyperendemic for DENV for many years [5,6,7].

Quantifying and predicting transmission of DENV can be difficult because of the number of variables needed to capture the nuances of transmission of just a single serotype. This becomes even more complex with 1) the interactions of more than one serotype, sometimes all four at once, and 2) when heterogeneity of individual variables due to environmental or sociological factors is included. However, investigations of these variables – either singly or in combination – can still provide valuable insight into the factors that drive local transmission patterns. One approach is vectorial capacity (VC), which is a measure of the intensity of transmission in a particular area by a mosquito population [8,9] and is defined as number of secondary infectious bites per day by a mosquito population given the presence of one primary infectious mosquito. The classical equation was modified for use in arbovirus systems by Garrett-Jones [10] is:

VC = \frac{{m{a^2}{p^N}b}}{{ - \ln \left( p \right)}}

Where *m* is the density of mosquitoes related to the human population, *a* is the man biting rate, *p* is the probability of daily survival of the mosquito, *N* is the extrinsic incubation period (the time required for a mosquito to become infectious given exposure), and *b* is the vector competence (the intrinsic permissiveness of a vector to infection, replication, and transmission of a virus) [11]. This equation has been used to study transmission and evaluate differences in parameter values, but few studies have used vectorial capacity as a means of comparing the transmission potential of site-specific, natural populations of *Aedes sp* [12]. Thus, this study will explore the utility of this approach to capture the patterns of transmission of DENV in hyperendemic areas by using a further modification of this equation, cumulative vectorial capacity (cVC), which takes into account the temporal interactions of vector competence and mosquito mortality among virus and/or mosquito populations [13].

The entomological and epidemiological patterns of three Colombian cities were described previously [14]. Among the main findings of this study was a lack of consistent correlation between mosquito density, observed infection rates of DENV in the mosquito populations of the three cities, and observed human DENV incidence. For example, the highest mosquito DENV infection rates were observed in Bello, which also had the lowest peak incidence of DENV infected humans. Interestingly, DENV incidence of Bello was observed to be similar to that of Riohacha, the city with the highest mosquito density as measured by the Breteau index (BI). However, the city with the highest DENV incidence – Villavicencio – showed lower BI than Riohacha [14]. In summary, this study demonstrated that entomological estimators of mosquito abundance do not readily capture patterns of DENV transmission observed in either the mosquito or human populations and so other factors must contribute to these observed differences [15,16,17,18]. Thus, this current study investigates whether quantification of additional parameters – site-specific vector competence and man-biting rate of the mosquitoes from those sites – better correlate to observed DENV transmission patterns.

## Materials and Methods

### Mosquito Rearing

Immature stages of mosquito were collected from samplings performed between mid-2012 and 2013 in the Colombian cities of Riohacha, Bello, and Villavicencio. Details about samplings, climatic and entomological characteristics are provided in Peña-García et al., 2016 [14]. Briefly, individual larva were collected and taken to the laboratory of the Biología y control de Enfermedades Infecciosas (BCEI) group at the Universidad de Antioquia. Larva were reared at standard conditions and fed with fish food (Truchina®, Cargill, Agribrands Purina Colombia S.A.) until adults emerged. Adults were blood-fed with chicken blood and sugar *ad libitum* to raise colonies. Adult mosquitoes were maintained in controlled conditions in the laboratory at temperature of 28°C and relative humidity of 70–80%. For experiments, generation F3 of field mosquitoes were used.

As a control population, laboratory maintained mosquitoes belonging to Chetumal colony were raised and maintained under same conditions described above at the Arthropod-Borne and infectious disease laboratory of Colorado State University [11,12,13,14,15,16,17,18,19].

### Virus and infections

A strain of DENV-2 (Jamaica1409) belonging to the Asian/American genotype, the same genotype circulating in Colombia [20], was used to perform experimental infections on mosquitoes from the three studied cities and the control group. Infections with this virus strain on mosquitoes from colony Chetumal has been previously characterized in other studies [11,12,13,14,15,16,17,18,19], which offer the opportunity to validate the experimental procedure by comparing infection proportions to those reported in those studies.

Infections were conducted according to Sánchez-Vargas et al. [21] Briefly, virus was seeded at MOI of 0.01 into T-25 flasks containing a confluent monolayer of C6/36 cells. The virus was propagated for 14 days, and then supernatant was mixed with sheep blood in a proportion of 1:1 resulting in a mixture with viral titer at 2.1 × 10^6^ PFU/mL. Mosquitoes of 4–6 days post-emergence from the four groups were fed on the mixture by using an artificial feeder with circulating water at a temperature of 37°C. Fully engorged mosquitoes were separated and approximately 60 mosquitoes of each city were dissected at 4, 7, 11 and 14 days after exposure by extracting midgut and heads. Samples were divided in half to be processed by immunofluorescence assay (IFA) and plaque assay. Midguts designated to immunofluorescence were fixed with 4% paraformaldehyde for at least 12 hours at 4°C and heads were squashed onto slides and fixed with acetone: PBS (3:1) for 30 minutes at –20°C. Samples processed by plaque assay were stored at –80°C until use.

### Quantifying Infection Kinetics within the Mosquito

To determine infection dynamics within the mosquitoes, IFA assays were performed with some modifications as done by Salazar et al. [19] Briefly, midguts were washed twice with 1x PBS-Ashburner’s solution-Tritón X-100 then incubated 90 minutes at 37°C with a solution of two different monoclonal antibodies: 3H5 (DENV-2 specific) and 4G2 (Flavivirus complex) to enhance detection. After two PBS washes, samples were incubated with a solution of anti-mouse antibody conjugated with biotin containing 0.01% Evans blue. After 90 minutes of incubation, samples were washed and incubated with a solution of streptavidin-fluorescein at 37°C by 60 minutes. Midguts were placed onto slides covered with Mowiol solution to fix samples and covered with cover slides. Detection of fluorescence was made on a fluorescence microscope. Heads were washed twice with PBS and stained using the same procedure as midguts. Infection and dissemination of DENV-2 were evaluated using logistic regression models using a quasi-likelihood link function to account for over-dispersion of the data at days 4 and 7 post-exposure. Analyses yield the odds of getting a positive midgut/head in each field-caught populations (Riohacha, Bello, Villavicencio) compared to the colony mosquitoes (Chetumal) per day post-exposure.

### Estimating body and head titer in positive mosquitoes

In addition, we used plaque assays on samples of midgut and head samples to titer the virus present in those tissues. The experiments were conducted following Sánchez-Vargas et al. [21] procedures. In these experiments, midguts and heads were homogenized onto 500 μL of DMEM media supplemented with 2% FBS and filtered. Serial dilutions of samples were prepared and 150 μL were added on monolayer of LLC-MK_2_ in 24-wells plates. After 1 hour of infection, cells were overlaid with agar-nutrient mixture (medium 199 1X, fetal bovine serum 7%, NaHCO_3_ 0,3%, Hank’s balanced salt solution with dextran 2%, DEAE 1%, MEM Vitamins 0,5X and MEM aminoacids 0,25X) and incubated at 37°C, 5% CO_2_ for 10 days. Plaques were counted after plates were incubated overnight with MTT 3 mg/mL solution. Data are reported as plaque forming units (PFU) per mL.

### Measuring Man-biting Rate of the Three Populations

To estimate this variable, we developed a new methodology by hypothesizing that the probability of a single mosquito feeding depends on the day after emergence of that mosquito. Specifically, we hypothesized that there was a greater likelihood of blood feeding at later days after emergence. Thus, we evaluated the likelihood of blood feeding of female mosquitoes at 1, 3, 5, and 6 days after emergence on healthy volunteers. Adult female mosquitoes were supplied with water and sugar *ad libitum* at all times and not starved prior to blood meal assessment.

Five non-infected/non-exposed females of the same age were released in a cage of 0.43 m^2^ (120 cm long, 53 cm width and 68 cm tall). A healthy volunteer introduced an arm in the cage for 15 minutes based on observations from Canyon and Hii (1997) [22]. During this time, the number of mosquitoes that fed and the time lapsed during feeding was recorded. The procedure was done with five volunteers for each of the three studied populations.

In order to obtain a model for the bite probability of mosquitoes through time, a logistic model was fit to the explanatory variable of time (given as days) to represent days post emergence. The resulting model was further used to predict the day at which 99% of mosquitoes would have fed given simultaneous emergence. The total man-biting rate was estimated by calculating the area under curve resulting from the model by using a regular trapezoid method through to 14 days. This value (area under the curve) was divided by 14 to obtain an average bites per day, but does not take into account mosquito age. Thus, our final model assumes that mosquitoes at all ages are circulating at the same time with a constant (average) biting rate.

In addition, we estimated the time (in minutes) that an individual mosquito takes to bite. This was done by recording time since introduction of the volunteer’s arm in the cage until a mosquito introduces its proboscis into the skin. This variable was called “time to bite” and was analyzed using Kruskal-Wallis test to check if there were differences among volunteers or populations. Also, we recorded the time since introduction of proboscis in the skin until it was removed; this variable was called “total feed time”. The “total feed time” data was log-transformed to be analyzed by ANOVA. We analyzed these variables in order to find differences among populations and to analyze if volunteer has a differential effect on bite.

### Estimation of Mosquito Density and Probability of Daily Survival

Estimates of mosquito density are taken directly from fieldwork data reported by Peña-Garcia and co-workers in 2016 [14]. In this work, authors reported number of mosquitoes captured at different times in houses from four neighborhoods. We estimated the average number of mosquitoes per house and the density of mosquitoes of each neighborhood at house level. We then combined measures of each house for the four neighborhoods and calculated an average for the entire city.

The average number of mosquitoes per house was then used to obtain a ratio of mosquitoes per person as an estimate of mosquito to human host density [23]. To do this, we obtained data from the projections of last nationwide census of human population developed by the Departamento Administrativo Nacional de Estadística (DANE) in 2005 (https://www.dane.gov.co/index.php/estadisticas-por-tema/demografia-y-poblacion/proyecciones-de-poblacion). DANE published statistics at the city level, including average number of inhabitants per house. Using this data, a ratio of mosquitoes and people per house (house-hold level) was obtained for each of the three cities.

Because there was such a disconnect in observed mosquito density and DENV incidence, we further investigated the sensitivity of our measures as relates to mosquito density and transmission by consideration of two density values in vectorial capacity: 1) Mosquito density estimated as described before in this paper (section Estimation of mosquito density) and 2) taking a value of 1, which results in nullifying the mathematical contribution of the variable [14].

Life tables for the three mosquito populations were constructed in parallel (Pérez L et al., in preparation). The number of adult mosquitoes surviving each day along the entire adult survival curve was used to estimate a probability of mosquito to survive each day. The total probability of daily survival was estimated by averaging the probability of all days evaluated. Data of life tables from each city were developed at two different temperatures, we averaged both datasets to obtain a unique value per population.

### Effective vector competence (EVC) and cumulative vectorial capacity (cVC) estimation

Since plaque assays quantify infectious viral particles, we used plaque assay data from head tissues at four studied days to determine effective vector competence (EVC) according to a modification of the methodology developed by Christofferson and Mores [13] and posterior estimation of cumulative vectorial capacity (cVC). This methodology uses the rate of mortality to estimate the change in vector competence through time and give a cumulative measure of vector competence called EVC. The EVC is then used in place of the static vector competence parameter to estimate cVC [13].

We modified the EVC methodology by fitting vector competence values at day post exposure to a logistic regression instead of a linear function. Thus, the function we used follows the form:

b\left( N \right) = \,\frac{1}{{1 + {e^{ - \left( {{\beta _1}N + {\beta _0}} \right)}}}}

Where *β*_1_ is the change in vector competence (*b*) per unit change in time (days, *N*) (slope of the line); and *β*_0_ is the y-intercept. The subsequent iterative calculations include the estimation of area under curve taking just the final two time points, which include the cumulative proportions of competent mosquitoes. Thus, the EVC was estimated taking an upper limit of *z* and a lower limit of *z*-1. Therefore, EVC is defined as [13]:

EVC = \varphi = \int_{z - 1}^z {{p^N}\left( {\frac{1}{{1 + {e^{ - \left( {{\beta _1}N + {\beta _0}} \right)}}}}} \right)dN}

And consequently, the estimation of cVC is defined by the following equation:

cVC\,\, = \,\,\frac{{m{a^2}\varphi }}{{ - \ln \left( p \right)}}

Where *φ* represents EVC and the other parameters are the same of the original formula previously explained.

### Ethics

Ethical Approval (Act N° 13, 29/07/2015) was obtained from the Bioethics Committee of the University of Antioquia, Medellin, Colombia.

## Results

### DENV-2 vector competence

As a control group, we determined that Chetumal colony mosquitoes had high mid-gut infection rates as determined by IFA: 48.1%, 79%, 60%, and 63% at 4, 7, 11 and 14 days post-exposure (dpe), respectively (Table 1). This is similar to rates demonstrated previously and demonstrates biological reproducibility in our methods [19]. Similar mid-gut infection rates were noted in mosquitoes from Riohacha (33.3%, 46.6%, 60%, and 50% at 4, 7, 11, and 14 dpe, respectively). Mosquitoes from Bello had higher initial infection rates (56% at 4 dpe) and maintained consistent rates (range 48.1–56%) over the time period sampled (Table 1). Villavicencio had relatively high infection rates (range 56–58.3%) until 11 dpe, but we observed a considerable drop in midgut infection rate at 14 dpe (20%).

**Table 1:** Midgut infection percentages of mosquitoes from the three field-caught origins and one laboratory colony and the log-odds of getting a positive midgut compared to the baseline of colony origins (Chetumal colony) at 4, 7, 11 and 14 days post-infection (dpi) according to IFA.

	Percentage of midgut Infection (n)				Log-Odds			

dpi	4	7	11	14	4	7	11	14

Chetumal	0.48 (27)	0.79 (29)	0.6 (30)	0.63 (27)	.	.	.	.
Riohacha	0.33 (27)	0.47 (30)	0.6 (30)	0.5 (30)	–0.148	–0.326*	<0.0001	–0.13
Bello	0.56 (25)	0.48 (33)	0.53 (28)	0.48 (27)	0.079	–0.308*	–0.0064	–0.15
Villavicencio	0.58 (26)	0.56 (25)	0.58 (24)	0.2 (30)	0.095	–0.233	–0.016	–0.43*

* statistically significant at 95% confidence.

We found that at 4 dpi, there was no significant difference in the log-odds of getting a positive midgut or head between any of the cities and the colony mosquitoes (Tables 1 and 2). At 7 and 14 dpi, there were differences in the log-odds of midgut and head positivity among at least one field caught population and Chetumal, while there was only a difference in head positivity at 11 dpi (Tables 1 and 2).

**Table 2:** Head infection percentages of mosquitoes from the three field-caught origins and one laboratory colony and the log-odds of getting a positive head compared to the baseline of colony origins (Chetumal colony) at 4, 7, 11 and 14 days post-infection (dpi) according to IFA.

	Percentage of head Infection (n)				Log-Odds			

dpi	4	7	11	14	4	7	11	14

Chetumal	0.03 (30)	0.2 (30)	0.5 (30)	0.63 (30)	.	.	.	.
Riohacha	0 (30)	0.13 (30)	0.4 (30)	0.47 (30)	–0.033	–0.067	–0.1	–0.167
Bello	0 (30)	0 (30)	0.07 (30)	0.17 (30)	–0.033	–0.2*	–0.43*	–0.467*
Villavicencio	0.03 (30)	0 (30)	0.2 (30)	0.23 (30)	<.0001	–0.2*	–0.3*	–0.4*

* statistically significant at 95% confidence.

### Correlation between Infection and Dissemination Rates

**Figure 1:**
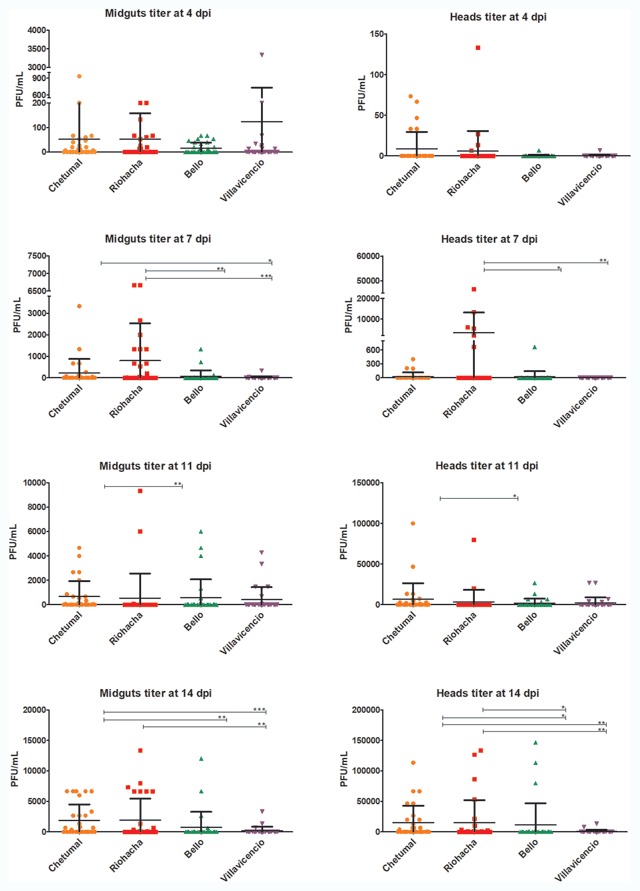
Viral titers at 4, 7, 11 and 14 days post-infection in the midgut and head tissues of mosquitoes from Riohacha, Bello, Villavicencio and the control population of Chetumal. Number of processed mosquitoes per city per dpi equals to 30. Significant differences are represented by *(*p* < 0.05), **(*p* < 0.01) and ***(*p* < 0.001). PFU/mL: Plaque forming units per milliliter.

Pairwise comparisons among population viral titers in the midgut and head tissues reveal that the highest discrepancies were between Riohacha and the populations of Bello and Villavicencio, where Riohacha had higher viral titers, most notable at 7 and 14 dpi (Figure 1).

All three cities had significant Pearson correlation coefficients (Riohacha, Pearson = .55; Villavicencio, Pearson = .51; Bellow, Pearson = .45), which suggests that the susceptibility of all three populations to developing a disseminated infection was relatively predicted by the ability to become infected.

### Differential Man-biting Rate among Mosquito Populations from the Three Cities

A Kruskal-Wallis test did not find significant differences in “time to bite” among cities (*X*^2^ = 2.61, df = 2, *p* = 0.27) or volunteers (*X*^2^ = 8.02, df = 4, *p* = 0.09). Similarly, no differences were found when we compared the “total feed time” among cities (F = 2.05, *p* = 0.13) or volunteers (F = 0.46, *p* = 0.76).

However, a significant difference in the frequency of successful biting among populations from the three cities was observed through Kruskal-Wallis test (*X*^2^ = 9.65, df = 2, *p* < 0.01). Paired comparisons through Wilcoxon rank sum test show significant differences between Bello and Riohacha (W = 92, *p* < 0.01) and Bello and Villavicencio (W = 276, *p* < 0.05) but not between Riohacha and Villavicencio (W = 160.5, *p* = 0.48).

**Figure 2:**
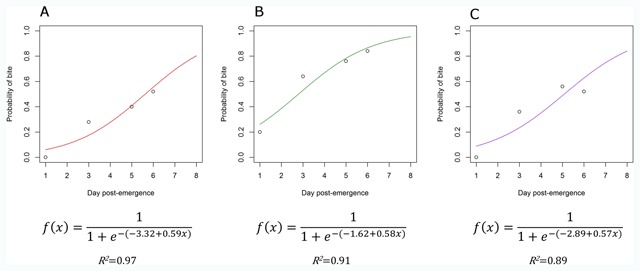
Curves and mathematical functions fitted to bite data from Riohacha **(A)**, Bello **(B)** and Villavicencio **(C)**. Every dot is the average of five volunteers. Mathematical functions and determination coefficient is shown under respective graph.

When the data were fitted to a logistic model, the average estimated bites per day was higher in Bello (0.76 bites per day), followed by Villavicencio (0.62 bites per day), and Riohacha (0.59 bites per day) (Figure 2). The estimated day post-emergence at which 99% of mosquitoes should feed was 11 days post emergence for Bello and 14 days post emergence for Riohacha and Villavicencio.

### Density and probability of daily survival

Bello had the lowest BI as observed previously [14] and this study showed Bello also had a lower average number of mosquitoes per house (0.62 mosquitoes per house). Villavicencio had a higher average number of mosquitoes per house (1.6), while Riohacha had the highest average number (1.88).

To obtain updated values of mosquito density, each of these values was divided by the respective average number of people per house as reported by DANE (4.5 for Riohacha, 3.8 for Bello and 3.7 for Villavicencio). Thus, the vector to host ratio at household level of Riohacha, and Villavicencio were quite similar at 0.42 and 0.43, respectively. However, Bello was estimated to have a lower ratio at household level of 0.16 mosquitoes per person.

The probability of daily survival was observed to be similar among the three mosquito populations. Riohacha showed marginally lower survival with a probability of daily survival of 0.9295, while Bello and Villavicencio had similar values of 0.9304 and 0.9306, respectively.

### Effective vector competence (EVC) and cumulative vectorial capacity (cVC)

**Figure 3:**
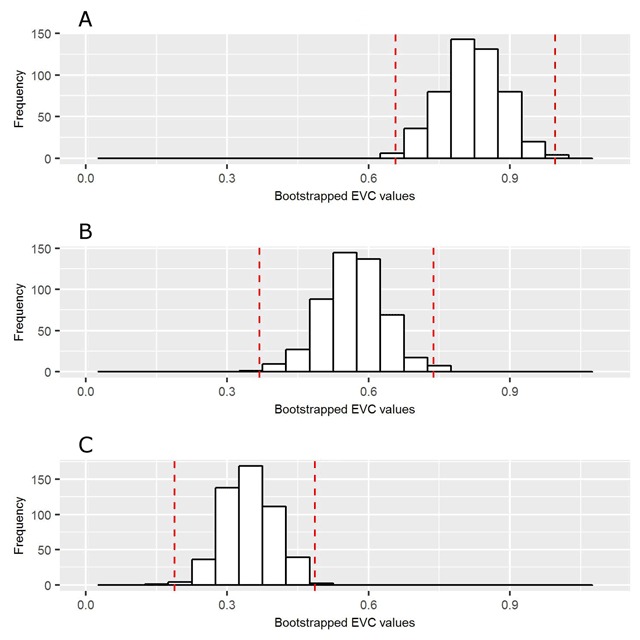
Distribution of bootstrapped EVC values for mosquitoes from Riohacha **(A)**, Bello **(B)** and Villavicencio **(C)**. Red lines indicate percentiles 2.5 and 97.5.

When we calculated the EVC for each population, our results indicate that the highest effective vector competence was in the mosquito population from Riohacha (0.82), followed by Bello (0.57), and finally Villavicencio (0.34) (Figure 3). Results of this modeling were statistically different when populations were pairwise compared through Tukey HSD test (ANOVA: F = 7631, *p* < 0.0001, HSD: *p* < 0.001 for all comparisons).

**Figure 4:**
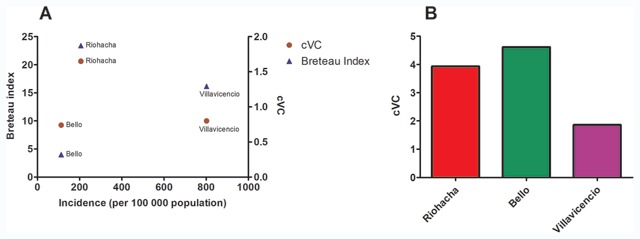
The role of density in cVC estimation. **(A)** Similarity of pattern produced by Breteau indices of the cities and cVC when density estimated is included related to dengue incidence during time study (mid-2012 to December 2013). **(B)** cVC estimated with vector density fixed at 1 showing higher values for Bello population.

When we combined all of the site-specific parameters (Table 3), there was no substantial difference noted and patterns followed that of the Breteau indices when plotted against human DENV incidence (Figure 4A). Riohacha had the highest cVC, followed by Villavicencio and Bello.

**Table 3:** Parameters used to estimate cVC of each of the three cities.

City	EVC (*φ*)*	Avg. bites per day (*a*)	Density (*m*)	Probability of daily survival (*p*)

Riohacha	0.82	0.59	0.42	0.9295
Bello	0.57	0.76	0.16	0.9304
Villavicencio	0.34	0.62	0.43	0.9306

* Head positivity by plaque assay used as a proxy for transmission potential of DENV-2.

### DENV Mosquito Infection Rates and Mosquito-density Independent Measures of cVC

In a previous study, we demonstrated that mosquito infection rates of natural populations were highest in Bello, followed by Villavicencio and Riohacha [14]. However, as demonstrated in Figure 4A, this is not explained by the either the EVC or cVC measures, nor the previous BI observations. Given the disparity in relative cVC estimates calculated above, observed mosquito density, and observed human incidence of DENV in the three cities, we investigated whether cVC would better predict or explain incidence if density were neutralized by holding the parameter at a value of 1. These density-independent values resulted in Bello having the highest cVC value followed closely by Riohacha and, with the lowest value, Villavicencio (Figure 4B). Thus, site-specific estimates of cVC that include biting rates, vector competence, and mosquito probability of daily survival are sufficient to capture observed infection rates in mosquito populations of the three cities, and mosquito density may confound predictions.

## Discussion

Measuring the kinetics of arboviruses in *Ae. aegypti* populations is a complex combination of biological, environmental, and sociological factors, and often do not follow observed differences in human incidence. For example, national data observed highest incidence Villavicencio, followed by Riohacha and Bello (which had similarly lower numbers) [14]. However, human incidence does not account for asymptomatic or subclinical cases and cannot be reliably used to evaluate total transmission.

It is worth noting that since the development of vectorial capacity concept, several modifications of the original equation has been developed [25], including studies that have utilized the vectorial capacity framework for estimations of transmission among natural populations of *Aedes sp* [12,13,14,15,16,17,18,19,20,21,22,23,24,25,26]. However, site-specific estimates based on data from corresponding mosquito population traits are few. Thus, this study was designed to determine whether site-specific formulations of EVC and cVC could recapitulate differences in mosquito infection rates previously published from the three cities [14]. Initially, we determined that vector competence rates were moderate in Bello where the highest infection rate was observed. However, this moderate vector competence was rescued by an enhanced biting rate, a known critically important driver of transmission [27]. It is important to note that the relative prediction of getting a disseminated infection in mosquitoes is important as vector surveillance is largely based on whole-body infection status, and thus understanding the dissemination kinetics in relation to infection kinetics can help put surveillance results into a larger context.

Further, when we used site-specific density estimates, we found that our measures did not follow that of observed field infection rates and that density-independent measures of cVC more accurately reflected field observations. This could suggest that a subset of mosquitoes is responsible for the majority of transmission events that is not readily captured in high-level measures of population density. We hypothesize that there is a critical threshold of transmitting mosquitoes necessary for virus perpetuation, but the factors that define this subset of mosquitoes has yet to be determined. It is critical that the role of density and the subset of infectious mosquitoes be further explored to add precision to future estimates and predictions of transmission. This would have obvious implications for vector control strategies that include population control (by insecticide spraying) versus in-door mosquito targeting, and may explain the disconnect between vector control implementation and a lack of decrease in dengue in some instances [28].

In addition, comparisons of site-specific transmission patterns of DENV-2 can be captured through the use of a mosquito density-independent cVC measure, and reconfirm that biting rate of the mosquito populations is an important and critical driver of transmission. Traditional approaches to measuring the man biting rate involve either indirect measures (human landing rates) [29] or a risk of exposure to human volunteers and we offer herein a method by which site-specific biting rates can be measured in controlled, low-risk ways [30]. In addition, several studies suggest that the distribution of *Ae. aegypti* is highly focal [31,32,33,34] and that biting rate is highly heterogenous even within a relatively small geographical area. This underscores the need for measuring man biting rates as a biological property of local mosquito populations.

The reasons why the mosquito population of Bello have higher biting rates compared to the other populations are unknown. Possible explanations include a lack of intraspecies competition for breeding sites close to domiciles, as well as the hosts themselves [35]. Regardless, this result does correlate with higher potential mosquito infection rates (cVC) as well as historical data that demonstrated infection rates higher in Bello than Riohacha and Villavicencio [14].

Our study is not without limitations. First, the use of a laboratory strain of DENV-2 cannot account for site-specific heterogeneity of the viral population, as there is vector competence variability both within and among strains and serotypes [36,37]. However, our study focuses on the mosquito-intrinsic characteristics affecting transmission, and so use of a laboratory strain (of the same genotype circulating in Colombia) does not invalidate our results. Indeed, likely our data supports the hypothesis that transmission is probably driven by factors associated with intrinsic mosquito characteristics and human-mosquito contact at least as much as it is by the efficiency of the specific viral strains circulating [38,39].

The transmission system of dengue virus is multifactorial, where numerous factors influence the probability of infection (e.g. social factors, environmental conditions) [24]. While it is important to take into account the interrelatedness of these many factors, it is also important to test hypotheses about individual and combinations of variables to isolate specific effects that may drive observed patterns of transmission and/or incidence. To that end, this study focused on key biological factors governing dengue transmission at the local level. However, we do acknowledge that other factors affect vectorial capacity, transmission, and observed incidence of dengue, such as temperature [40,41]. Indeed, heterogeneity in these other factors might result in differences in transmission, and future studies should continue to investigate these factors individually and in combination to determine synergism or antagonism among variables and comprehensively describe the transmission systems of arboviruses.

In summary, these results offer insights into the complex transmission patterns in three Colombian cities. We offer a refined methodology for estimating the site-specific man-biting rate of focal mosquito populations. We also demonstrate the necessity of estimating vector competence from field-caught mosquitoes by demonstrating intraspecific heterogeneity from relatively proximal communities, as well as explore circumstances under which site-specific consideration of mosquito density may not be as necessary. Ultimately, we demonstrate that studies of *Ae. aegypti* vectorial capacity should be made in affected areas whenever possible to assess variability in these parameters for more geo-specific assessments of the force of infection and transmission of DENV in mosquito and human populations.
